# AMF-mediated rhizospheric interactions by soil microbiota and metabolites in intercropping of tobacco and maize to regulate the soil nutrients

**DOI:** 10.3389/fpls.2025.1683474

**Published:** 2025-11-27

**Authors:** Di Liu, Shulei Liu, Peiyan Zhao, Fuzhao Nian, Junying Li, Ningbo Han, Shanqin Yang, Jialong Yu, Xiaopeng Deng, Yating Liu

**Affiliations:** 1 College of Tobacco Science, Yunnan Agricultural University, Kunming, China; 2 Yongsheng County Branch of Lijiang Tobacco Company, Lijiang, China; 3 Yunnan Academy of Tobacco Agricultural Sciences, Kunming, China; 4 Weishan County Branch of Dali Tobacco Company, Dali, China; 5 Songming County Branch of Kunming Tobacco Company, Kunming, China

**Keywords:** tobacco, species interaction, arbuscular mycorrhizal fungi, microbial diversity, rhizosphere metabolites

## Abstract

**Introduction:**

Colonization of arbuscular mycorrhizal fungi (AMF) can form the root symbiotic network of co-cultured plants roots and hyphae thus promote plant growth. Maize is often intercropped during the harvesting period of tobacco in tobacco-growing areas in China. AMF Colonization has been shown to be an effective approach for regulating the synergistic growth of Nicotiana tabacum and maize.

**Methods:**

In this study, pot experiments were conducted, and samples were analyzed using 16S rDNA and ITS high-throughput sequencing for bacteria and fungi, respectively,and LC-MS/MS widely targeted metabolomics of soil. Differences in microbiota and metabolites in the rhizosphere soil of tobacco and maize, as well as their correlations with the agrochemical properties of soil at the vegetative stage of crop growth, were investigated under AMF colonization to clarify the feedback regulation of plant growth by AMF inoculation and rhizosphere interactions between Nicotiana tabacum and maize.

**Results:**

The results showed that the growth of tobacco and maize inoculated with AMF was better than that of uninoculated plants, and this was related to the enhancement of plant nutrient uptake by AMF and root interactions between the two crops, which resulted in significant increases in the content of alkaline hydrolyzable nitrogen (N), available phosphorus (P), and available potassium (K) in the rhizosphere soil of tobacco. The stem girth of tobacco and the biomass of maize were significantly higher under intercropping than monoculture, as intercropping increased the relative abundances of Penicillium, Trichoderma, Blastomonas, and Sphingomonas in the rhizosphere soil of tobacco and maize; the abundance of Penicillium was higher in rhizosphere soil of AMF inoculated treatments. AMF inoculation and intercropping cultivation respectively led to the down-regulation of differentially expressed metabolites (DEMs) in the rhizosphere soil of tobacco and maize. Additionally, pH and organic matter are key environmental factors influencing soil microbial communities.

**Discussion:**

Overall, intercropping and AMF inoculation mediated rhizospheric interactions by soil microbiota and metabolites in intercropping can regulate plant growth and improving the content of N, P, and K in rhizosphere soil. Our findings provide new insights with implications for AMF application on interactions between the root systems of tobacco with maize or other plants during the tobacco harvesting period.

## 1 Introduction

During the process of crop cultivation, *Nicotiana tabacum* belongs to the genus *Nicotiana*, family Solanaceae, and long-term continuous cropping decreases microbial abundance and functional diversity and reduces soil quality in tobacco fields, which in turn affects the growth and yield of tobacco plants (Liu et al., 2023a; Pan et al., 2023). Maize (*Zea mays*) belongs to the genus *Zea* in the Poaceae family, and it is an important food and fodder crop. While a large number of studies have shown that root interactions between crops enhance species diversity, crop productivity (Zou et al., 2019), soil nutrients and the activity of related enzymes, microbial diversity in soil (Guo et al., 2019), and plant nutrient uptake and utilization (Zhu et al., 2023). And the intercropping of tobacco with maize during the tobacco-harvesting period can improve land utilization and increase economic benefits (Gao et al., 2023). Research shows that crop intercropping can enhance the diversity and abundance of bacteria and metabolites in soil (Shuangshuang et al., 2023). Such as the intercropping of soybean with maize can increase the uptake of nitrogen (N) and phosphorus (P) and soil microbiota diversity (Zhi-Dan et al., 2019). Sun found that the intercropping of *Ophiopogon japonicus* with maize can improve the fungal diversity of *O. japonicus* and decrease the fungal diversity of maize in rhizosphere soil (Sun et al. 2025a). Peanut and cotton intercropping can induce leaf photosynthesis, promote the uptake of nutrients such as N, P, and potassium (K) different organs of the crops., and enhance plant growth. In addition, research shows that the accumulation of root-specific secretions associated with L-tryptophan synthesis and microbial communities associated with carbon (C) sources and amino acid metabolism are also enriched in intercropping systems (Lu et al., 2024). Overall, intercropping of crops significantly affects growth and the soil microenvironment.

Researches indicates that arbuscular mycorrhizal fungi (AMF) are beneficial rhizosphere soil microorganisms that are widely distributed in terrestrial ecosystems, approximately 80% of terrestrial plants can form symbioses with arbuscular mycorrhizal (Berruti et al., 2016). AMF can transport resources such as nutrients and water after infecting the roots of different plants, which increases the effective use of nutrients and water by plants (Berruti et al., 2016; Sharma and Kayang, 2017; Wang et al., 2019; Liu et al., 2022). AMF also affects the allocation of resources among interplanted plants (Zhu et al., 2023), improves the productivity and sustainability of the faba bean–maize intercropping system, and modifies the abundance of microorganisms in rhizosphere soil under abiotic stress (Wang et al., 2019). Additionally, AMF can increase the yield and quality of plants by up-regulating the phenylpropanoid metabolic pathway (Kaur and Suseela, 2020), enhance the activity of antioxidant enzymes in plants, and promote the synthesis of secondary metabolites such as phytoalexin, glycoproteins, and phenolic compounds, which enhances plant disease resistance (Li and Zeng, 2020).

The interactions between tobacco and maize in the rhizosphere soil and their effect on plant growth merit increased attention because of their potential to promote the utilization of nutrients in plants; increase the content of N, P, and K in soil, and help overcome challenges associated with continuous cropping. In this study, we carried out pot experiments at the vegetative stage of maize and *Nicotiana tabacum* using crop varieties that are widely grown in Yunnan Province to elucidate interactions between crop roots and explore the effects of AMF and intercropping on plant growth and the soil microenvironment. We were particularly interested in examining the effects of AMF and intercropping on soil N, P, and K nutrients, the abundance and functions of microbial, and metabolites, as well as correlations among these characteristics. Our findings have implications for the use of mycorrhizal technology for analyzing plant–soil microorganism–metabolite associations, improving soil nutrient utilization efficiency, and promoting the growth of plants in intercropping systems.

## 2 Materials and methods

### 2.1 Experimental design

Pot experiments were carried out from March to June 2023 in the greenhouse of Yunnan Agricultural University. tobacco (K326, supplied by Yuxi Zhongyan Seed Co., Ltd.) was transplanted after 45 d of aseptic floating seedling cultivation, and maize (Yunrui 319, purchased from Yunnan Tianrui Seed Co., Ltd.) was aseptically budded 10 d before transplantation. The AMF of *Funneliformis mosseae* was propagated in maize roots for 12 weeks. The inoculum contained spores, mycelium, and colonized root fragments is approximately 2,000 spores per 50 grams of soil. and was propagated with sterile vermiculite and farmland soil using the “sandwich” method. The control treatment received an equivalent amount of autoclaved (121°C, 2 hours; Disinfect once every 24 hours, for a total of 3 times.) inoculum to account for any non-AMF microbial effects. The potting experimental culture medium was obtained by subjecting vermiculite and farmland soil to high-pressure steam sterilization. (The volume ratio of vermiculite to farmland soil is 1:3.), and the agrochemical properties were alkaline hydrolyzable N (AN), 86.4 mg·kg^-1^; available P (AP), 22.5 mg·kg^-1^; available K (AK), 328.57 mg·kg^-1^; organic matter(OM), 18.0 g·kg^-1^; and pH = 6.8 (n=3). In the experiment, tobacco and maize were monocultured or intercropped, with or without AMF inoculation, which permitted a two-factor analysis to be performed. Two plants were grown in each pot with 8 kg of sterile substrate, and there was a distance of 30 cm between each plant. Uncontaminated and uniformly grown maize/tobacco seedlings were selected and transplanted into the sterile substrate, the “sandwich” method was used to inoculate AMF around the roots of the plants (200 g per plant). In the non-AMF group, inoculations were performed with inactivated AMF, Fertilizer application was calculated based on the field standard for tobacco and maize (1100 plants per 667 m² applying 4.1 kg pure N), which was converted to a per-pot basis. each treatment was replicated 12 times (pots) in a completely randomized design. The methods for watering, weeding, and pest control methods were consistent in all treatments. After cultivation for about 30 days, the samples of corn and tobacco roots were stained using the acidic eosin-lactic acid glycerol staining system. The symbiotic development status of plant root arbuscular mycorrhizal fungi (AMF) was evaluated by the cross-observation method. Structures such as AMF hyphae, arbuscules, vesicles, and hyphal rings were observed, which were regarded as effective colonization.

### 2.2 Sample collection and testing

Soil and plant samples were taken at the vegetative stage after 45 days of transplanting to measure relevant indicators. There were 6 treatment groups. Samples were taken from both tobacco and corn, resulting in a total of 8 samples. (**Table 1**). The rhizosphere soil was collected by shaking the roots and removing impurities; the soil was then stored in micro-centrifuge tubes (10 mL) at -80°C. Another sample was air-dried and screened with a 2 mm sieve to determine soil agrochemical properties and enzyme activities. The roots of plants were washed, and excess water was absorbed; the fresh weight of the aboveground and underground parts was measured. Plant samples were killed at 105°C for 30 min, dried in an oven at 75°C until a constant weight, then measured the dry weight. The samples were ground and sieved through a 60-mesh sieve for the determination of nutrients in different organs of the plant.

**Table 1:** Sample names and descriptions.

Samples	Descriptions
TM-	Tobacco monoculture and not inoculated with AMF
TM+	Tobacco monoculture and inoculated with AMF
MM-	Maize monoculture without AMF inoculation
MM+	Maize monoculture with AMF inoculation
MI-/TI-	Tobacco intercropping with maize and not inoculated with AMF
MI+/TI+	Tobacco intercropping with maize and inoculated with AMF

TI and MI samples represent tobacco and maize plants cultivated together in the same container. Both were planted and harvested from a shared pot used for intercropping. However, the harvesting of each plant (tobacco and maize) and their respective rhizosphere soil was conducted separately.

#### 2.2.1 Determination of agronomic traits, photosynthetic parameters, and nutrient content of different organs in plants

Plant height, stem girth, leaf number, and maximum leaf length and width of tobacco seedlings were measured at 45 days of transplanting according to the “YC/T 142–2010 nicotiana agronomic traits survey and measurement methods.” The relative content of chlorophyll in leaves was determined using a portable chlorophyll meter (SPAD-502, Konica Minolta Sensing, Inc., Japan). Photosynthetic parameters such as the transpiration rate (Tr), stomatal conductance (Gs), net photosynthetic rate (Pn), and intercellular CO_2_ concentration (Ci) of the fourth (top to bottom) functional leaf of the same part of maize and tobacco were measured using a portable photosynthesis meter (GFS-3000). A leaf chamber with a red and blue light source was used to measure the photosynthetic parameters, and the light intensity was set at 1,000 μmol·m^-2^·s^-1^ (n=4) (Chen et al., 2024). The N, P, and K content of different organs above and below the ground in maize and tobacco were measured in different treatment groups (National Standard NYT2017-2011).

#### 2.2.2 Measurement of the AMF colonization rate in plant roots

The colonization rate of AMF (*F. mosseae*) was calculated using the acidic magenta staining method and crisscross method of fresh root samples (Liu et al., 2022).

Colonization rate of AMF (%) = (total number of view fields - number of blank fields)/total number of view fields×100%.

#### 2.2.3 Determination of agrochemical properties and enzymatic activity of rhizosphere soil

The soil respiration rate (RA) was determined using an SRS2000 T portable soil respirometer (Shen et al., 2023); soil pH was determined with the water leaching method. The content of organic matter in soil was determined with the potassium dichromate titration method, the content of AN in soil was determined with the alkaline diffusion method, AP in soil was determined with the molybdenum-antimony colorimetric method, and the content of AK in soil was determined with the flame photometric method. The specific methods were performed as described in “Soil Agrochemical Analysis” (Bao, 2005). Soil sucrase (SC), catalase (CAT), acid phosphatase (ACP), urease (UE), nitrate reductase (NR), and phytase (PAE) activities were determined using various kits (Suzhou Geruise Biotechnology Co., Ltd.) (Wang et al., 2022).

#### 2.2.4 Analysis of microorganismal diversity in rhizosphere soil

The bacterial and fungal diversity of soil microorganisms was determined by Shanghai Personal Biological Company. The soil was subjected to DNA extraction, PCR amplification, library construction, and 16S rDNA high-throughput sequencing. The universal primers for bacteria (F: 5′-ACTCCTACGGGAGGCAGCA-3′, R: 5′-GGACTACHVGGGTWTCTAAT-3′) were designed for PCR amplification of the 16S_V3V4a region of the bacterial 16S rRNA gene. The universal primers for fungi (F: 5′-GGAAGTAAAAGTCGTAACAAGG-3′, R5′-GCTGCGTTCTTCATCGATGC-3′) were designed for PCR amplification of the ITS_V1 region of the fungal ITS gene.

#### 2.2.5 Analysis of metabolites in rhizosphere soil

Soil metabolites were extracted and subjected to broadly targeted metabolomics analysis (Wuhan Metware Biotechnology Co., Ltd., China). Fifty mg soil samples were thawed on ice, homogenized with 500 μL of ice methanol/water (70%, v/v), and incubated on ice for 15 min, they were then centrifuged at 12,000 rpm for 10 min at 4°C, and 400 μL of the supernatant was placed into a centrifuge tube. Next, 500 μL of ethyl acetate/methanol (V, 1:3) was added to the precipitate, incubated on ice for 15 min after shaking for 5 min, and then centrifuged at 12,000 rpm for 10 min at 4°C. After 400 μL of the supernatant was collected, the two supernatants were mixed and concentrated. After drying, 100 μL of 70% methanol-water was added, sonicated for 3 min, and centrifuged at 12,000 rpm for 3 min at 4°C; next, 60 μL of the supernatant was extracted for UPLC-MS/MS analysis. Root and microbial exudates in soil were detected using an UPLC-ESI-MS/MS system (UPLC, ExionLC™ AD https://sciex.com.cn/) and Tandem mass spectrometry system (https://sciex.com.cn/). The analytical conditions were as follows, UPLC: column, Agilent SB-C18 (1.8 µm, 2.1 mm * 100 mm); The mobile phase was consisted of solvent A, pure water with 0.1% formic acid, and solvent B, acetonitrile with 0.1% formic acid. Sample measurements were performed with a gradient program that employed the starting conditions of 95% A, 5% B. Within 9 min, a linear gradient to 5% A, 95% B was programmed, and a composition of 5% A, 95% Bwaskept for 1 min. Subsequently, a composition of 95% A, 5.0% B was adjusted within 1.1 min and kept for 2.9 min. The flow velocity was set as 0.35 mL per minute; The column oven was set to 40°C; The injection volume was 2 μL. The effluent was alternatively connected to an ESI-triple quadrupole-linear ion trap (QTRAP)-MS

#### 2.2.6 Statistical analysis

Assess the effects of AMF and crop interactions on plant agronomic traits, biomass, photosynthetic indexes, nutrient content in different organs, soil agrochemical properties, and enzyme activities, the comparison between the two groups was conducted using the independent samples T-test; the comparison among the four groups was performed using the two-factor analysis. All data were expressed as mean ± SE. All statistical analyses were performed using SPSS (22.0, IBM Co., Armonk, NY, USA). Microorganism sequence data were analyzed using the QIIME2 and R software packages (v3.2.0) to characterize the diversity of bacterial and fungal amplified sequence variants (ASVs), and the common mycorrhizal network (CMN) was constructed using Gephi 0.10.1 for visualization and analysis. Differentially expressed metabolites (DEMs) in the soils of different treatments were analyzed on the Metware platform (https://cloud.metware.cn), key DEMs were screened and VIP was analyzed using a combination of analysis of variance and ordinary least squares regression analysis (*p* < 0.05 and VIP > 1). Environmental factor correlation analysis was used to investigate the effects of changes caused by microorganisms. Partial least squares path modeling (PLS-PM) was used to analyze the associations between rhizosphere soil nutrients, metabolites, and microorganisms, as well as the mechanisms underlying their effects on plant growth.

## 3 Results and analysis

### 3.1 Colonization rate of AMF in plant roots

**Figure 1:**
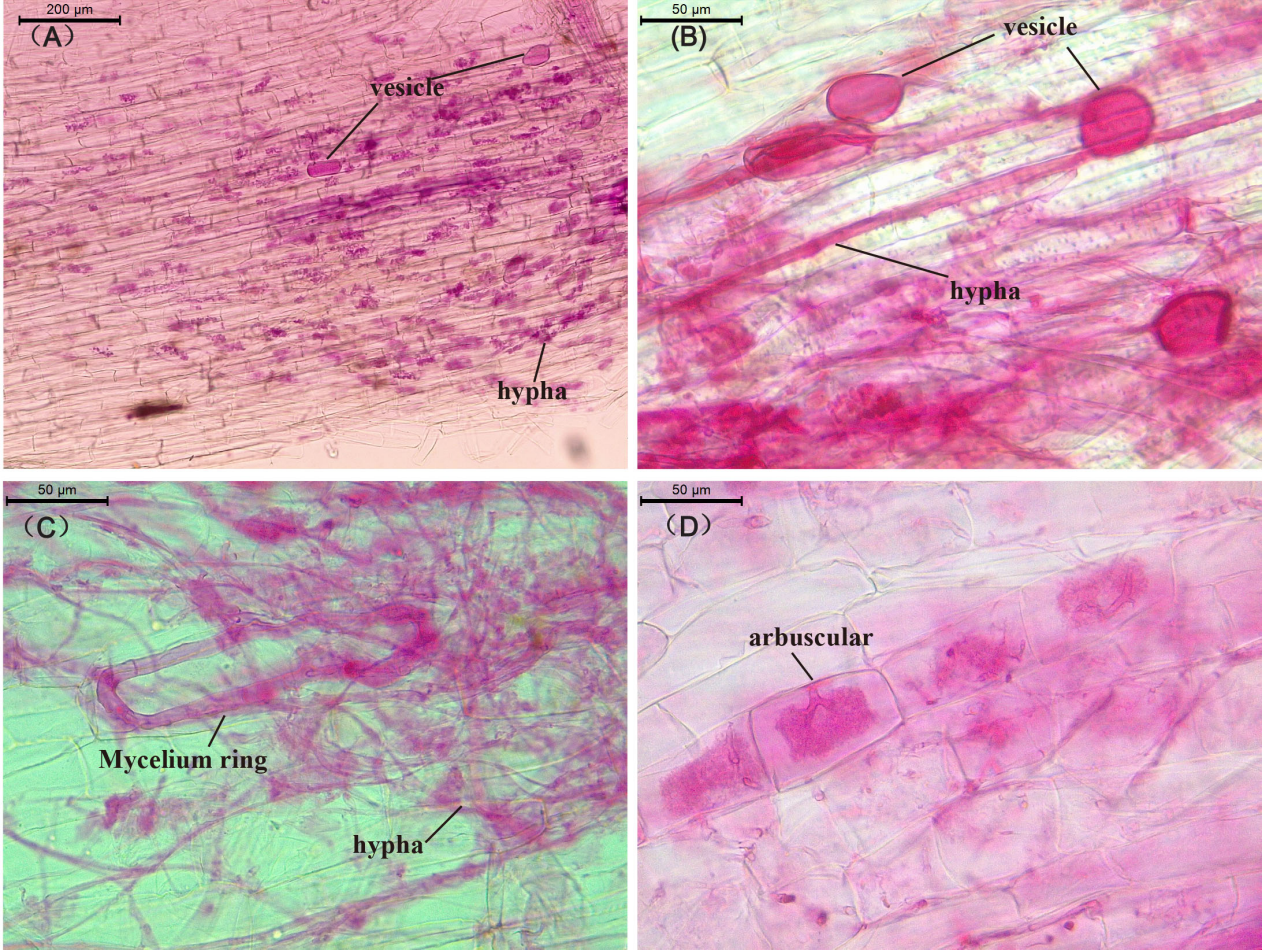
Optical micrograph of AMF-infected plant roots. **(A)** mycelial structure, **(B)** vesicular structure, **(C)** mycelial ring structure, **(D)** arbuscular structure, **(A)** shows the root structure of flue-cured tobacco, while **(B–D)** are the root structures of corn.

Under AMF inoculation, tobacco and maize roots were successfully colonized by a variety of AMF (*F. mosseae*) structures: hyphae, arbuscules, vesicles, and mycelia spores (**Figure 1**), the AMF colonization rate was above 80% (**Supplementary Table S1**). There was no significant difference in the colonization rate of AMF in the roots of tobacco and maize under monoculture and intercropping, and no AMF colonization was observed in the roots of uninoculated plants.

### 3.2 Effects of AMF and crop interactions on plant growth

**Figure 2:**
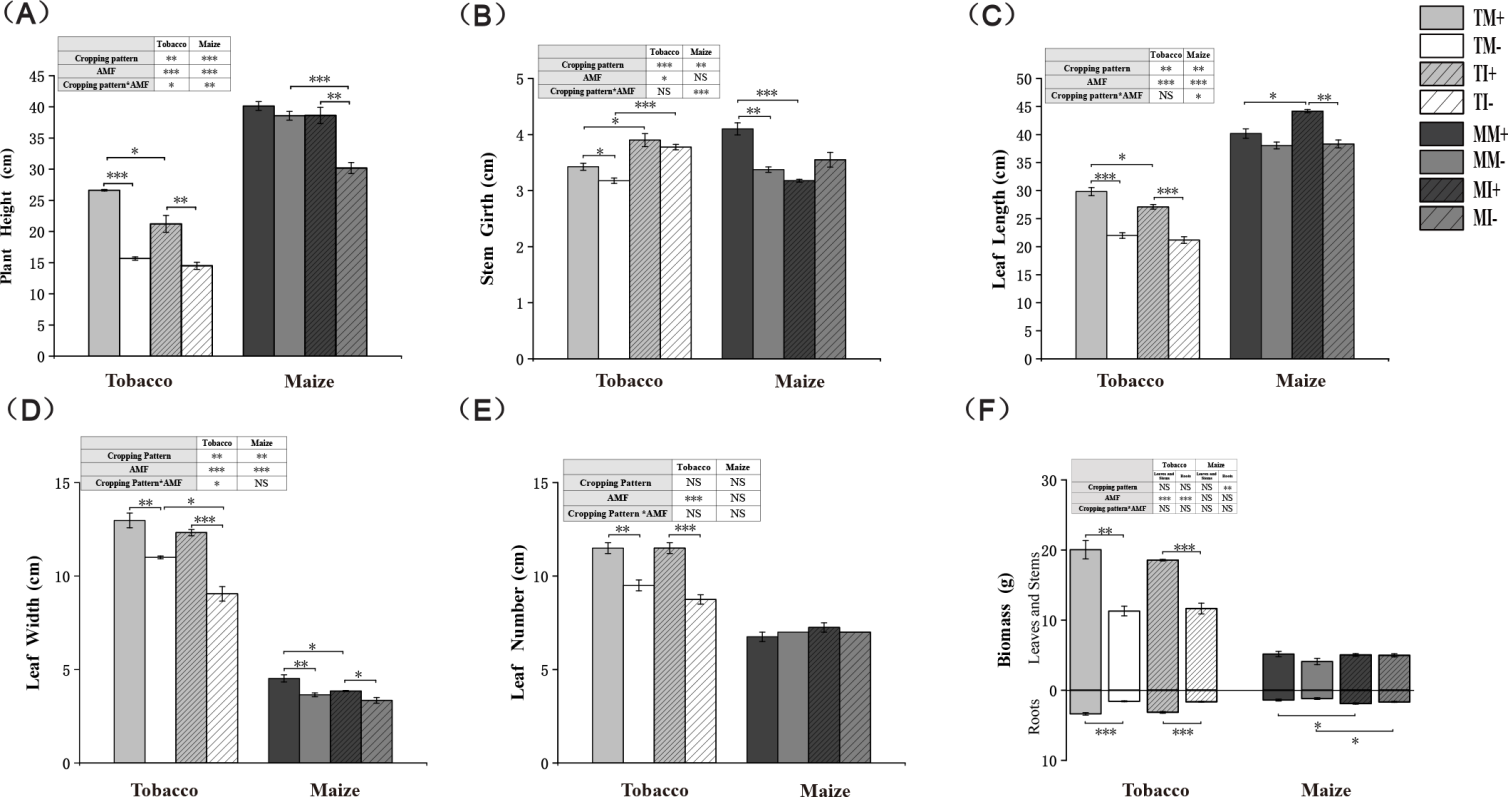
Agronomic traits and biomass of tobacco and maize in different treatment groups. * indicates significant difference between the two groups (*0.01≤ *p* < 0.05, **0.001≤ *p* < 0.01, ***p < 0.001, t-test; n=3). * In the table represents a two-factor analysis of the effects of planting patterns and AMF on agronomic traits or biomass of tobacco and maize (*0.01≤ *p* < 0.05, **0.001≤ *p* < 0.01, ***p < 0.001, two-way anova; n=3), the same below.

The plant height, leaf length, leaf width, and the number of leaves of tobacco and maize were significantly higher in AMF-inoculated crops than non-inoculated crops, The application of AMF resulted in a 77.5% increase in the above-ground biomass of tobacco in monoculture and intercropping conditions, and a 59.3% increase in the below-ground biomass. For corn, the above-ground biomass increased by 25.7% and 1.1% respectively, while the below-ground biomass increased by 18% and 13.8% respectively. The plant height and leaf width were significantly lower and the root biomass of maize and stem girth of tobacco were significantly higher under intercropping than monoculture. These findings confirm that AMF inoculation promoted the growth of maize and tobacco, and intercropping of tobacco and maize at the vegetative stage promoted plant growth (**Figure 2**). Two-factor analysis showed that crop intercropping significantly affected the agronomic characters of tobacco and maize. AMF inoculation significantly affected the plant height, leaf length, and leaf width of tobacco and maize and the biomass of tobacco. The interaction between AMF and planting pattern significantly affected the height of tobacco and maize and the stem girth and leaf length of maize.

The content of chlorophyll in leaves and the Pn of tobacco were significantly higher in the AMF-inoculated crops than non-inoculated crops (**Supplementary Figure S1A**) under monoculture (**Supplementary Figure S1E**). When plant roots were inoculated with AMF, the Tr rate and Ci were significantly higher (**Supplementary Figures S1B, C**) and the content of chlorophyll in tobacco leaves was significantly lower under intercropping of tobacco and maize than monoculture planting. Compared with uninoculated AMF treatments, the Tr, Gs, and Pn of tobacco and maize (**Supplementary Figures S1B, D, E**) were significantly higher under intercropping than monoculture. Two-factor analysis showed that the interaction between the planting pattern and AMF had significant effects on the content of chlorophyll in leaves and the Pn of tobacco and maize.

**Figure 3:**
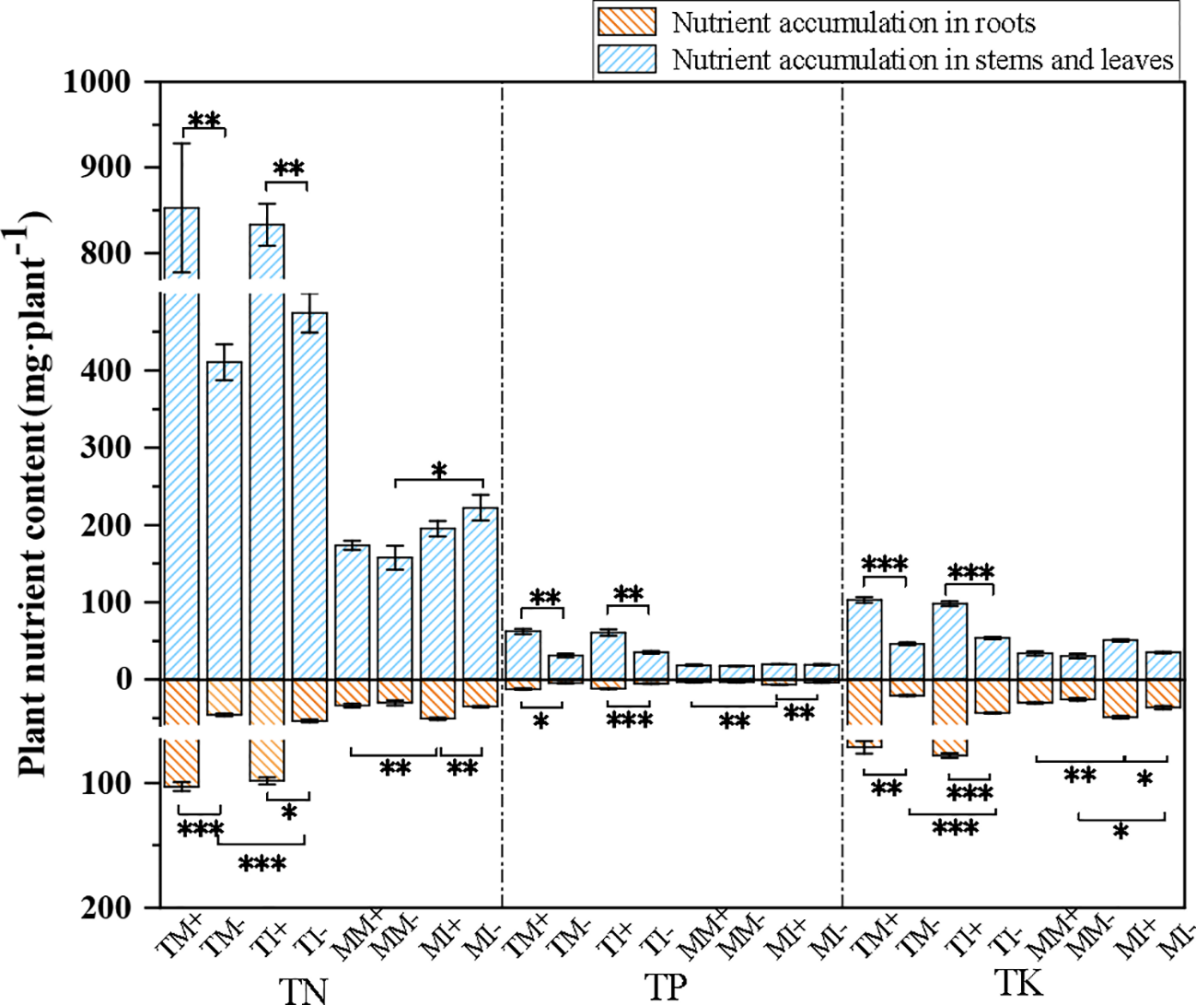
Plant nutrient accumulation of tobacco and maize in different treatment groups. Independent sample t-test for significant differences “* 0.01≤ p<0.05, **0.001≤p<0.01, *** p<0.001”.

Total N, P, and K accumulation in the aboveground and belowground organs of tobacco was significantly higher in AMF-inoculated crops than non-inoculated crops. Among them, the application of AMF increased the total nitrogen, phosphorus and potassium accumulation in the above-ground organs of the monoculture treatment tobacco by 107.6%, 100.6% and 95.7% respectively, and in the underground organs by 123.4%, 161.1% and 241.6% respectively. For the intercropping treatment, the total nitrogen, phosphorus and potassium accumulation in the above-ground organs of tobacco increased by 75.7%, 70.4% and 67.3% respectively; and in the underground organs, the total nitrogen, phosphorus and potassium accumulation increased by 82.8%, 124.0% and 79% respectively. Total N, P, and K accumulation was higher in tobacco and maize roots under intercropping than monoculture (**Figure 3**). The results showed that the interaction between AMF inoculation and planting pattern of tobacco and maize promoted nutrient uptake and accumulation in the crops.

### 3.3 Effects of AMF and planting pattern on crop rhizosphere soil nutrients, microbial diversity, and metabolomics

#### 3.3.1 Effects of AMF and planting pattern on crop rhizosphere soil agrochemical properties and enzyme activity

**Figure 4:**
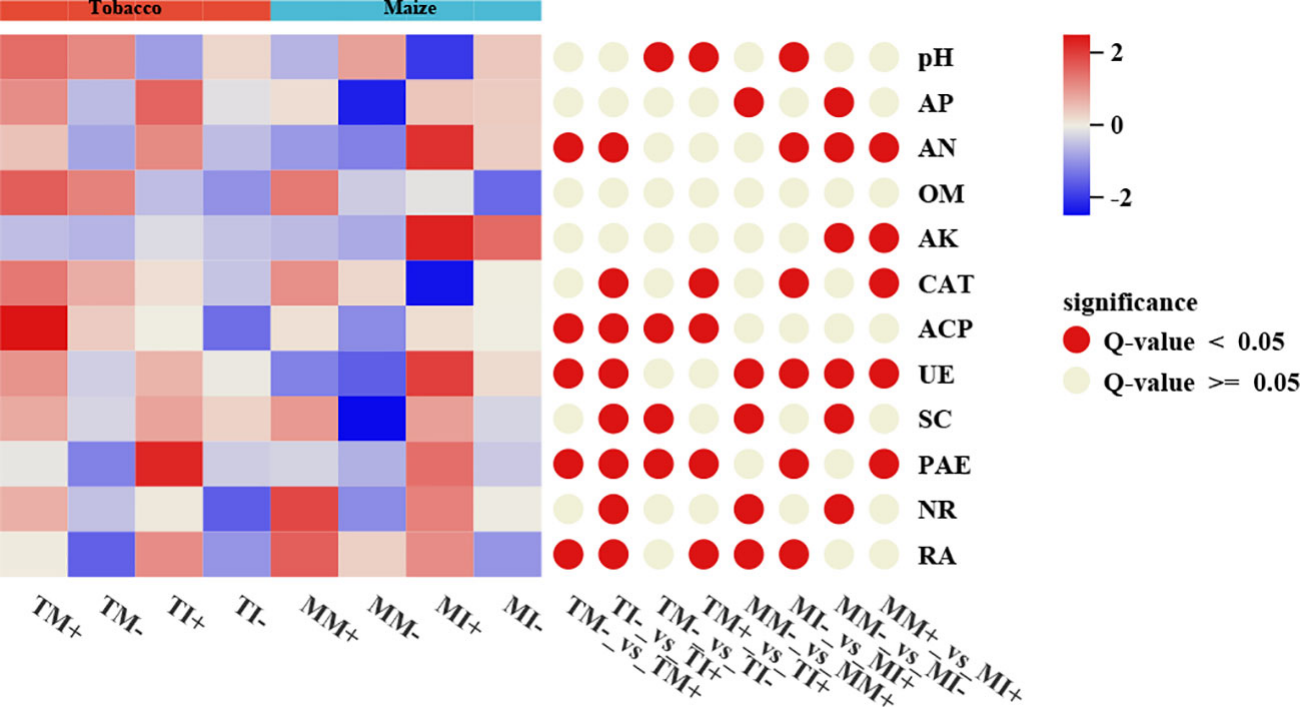
Physicochemical properties and enzyme activities of rhizosphere soil of tobacco and maize in different treatment groups. The red circle on the right indicates a significant difference between the two groups (p<0.05), and the gray indicates no significant difference.

The content of AN, activity of ACP, UE, PAE, and RA of rhizosphere soil of tobacco were higher in AMF-inoculated treatments than non-inoculated treatments, the RA of maize rhizosphere soil was also higher in the AMF-inoculated treatments than non-inoculated treatments. Compared with monoculture, soil pH and ACP activity were significantly lower in rhizosphere soil of tobacco, and the activity of UE and PAE, the content of AN and AK were higher in the rhizosphere soil of maize under intercropping mode (**Figure 4**). Overall, AMF inoculation and interactions between crops increased the content of N, K, and P content in the soil.

#### 3.3.2 Effects of AMF and planting pattern on the abundance and functional diversity of microorganisms in rhizosphere soil

##### 3.3.2.1 Effects of AMF inoculation and planting pattern on the community and structural composition of microorganisms in rhizosphere soil

**Figure 5:**
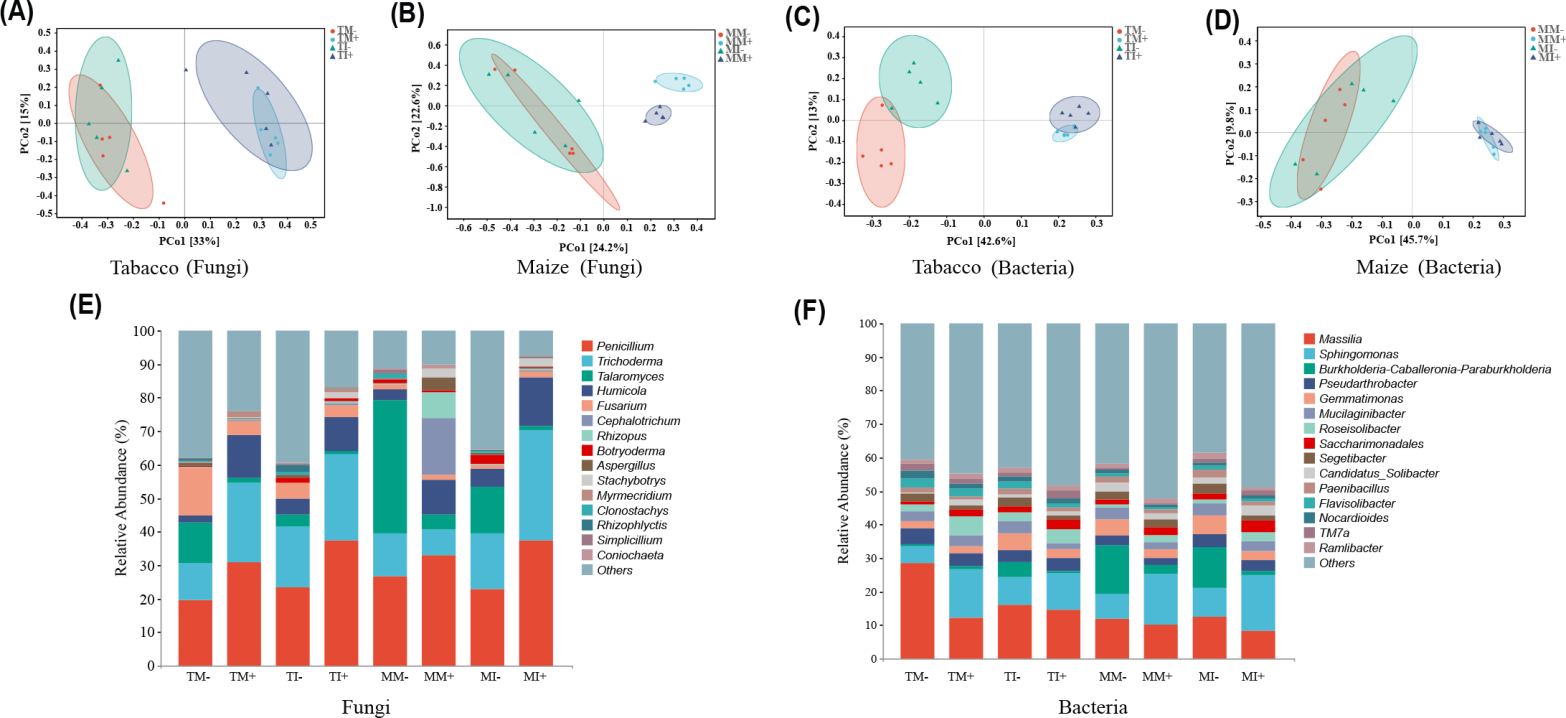
Analysis of rhizosphere soil microbial community structure and marker species of maize and tobacco under different treatments. [**(A)** PCoA analysis of tobacco fungal community; **(B)** PCoA analysis of maize fungal community; **(C)** PCoA analysis of tobacco bacterial community; **(D)** PCoA analysis of maize bacterial community; **(E)** Analysis of the level composition of fungi; **(F)** Bacterial level composition analysis].

Principal coordinate analysis of microbial community structure in soil based on ASV abundance showed that the PCo1 and PCo2 axes of the main components of fungi in tobacco rhizosphere soil explained 33% and 15% of the variation in the data, respectively (**Figure 5A**), and in maize rhizosphere soil explained 24.2% and 22.6% of the variation in the data, respectively (**Figure 5B**). The PCo1 and PCo2 axes of the main components of bacteria in the rhizosphere soil of tobacco explained 42.6% and 13% of the variation in the data, respectively (**Figure 5C**), and in maize rhizosphere soil explained 45.7% and 9.8% of the variation in the data, respectively (**Figure 5D**). Aggregation within each treatment was high, bacteria and fungi were dispersed in different treatments, indicating that AMF inoculation and planting pattern caused changes in community structure of bacterial and fungal in plant rhizosphere soil. The number of fungi ASVs specific to tobacco rhizosphere soil was higher under intercropping than monoculture. Compared with non-inoculated treatments, the number of fungi ASVs specific to maize rhizosphere soil was significantly higher and the number of bacteria ASVs specific to tobacco rhizosphere soil was lower with AMF inoculation treatments (**Supplementary Figure S2**). Analyses of the dominant communities and differences in the abundances of the 15 most abundant fungal and bacterial genera in maize rhizosphere soil indicated that the dominant fungi were *Penicillium* and *Trichoderma*. The relative abundances of *Penicillium* and *Trichoderma* in tobacco rhizosphere soil and the relative abundances of *Penicillium* and *Humicola* in maize rhizosphere soil were higher in AMF-inoculated treatments than non-inoculated treatments. The relative abundances of *Penicillium* and *Trichoderma* fungi in rhizosphere soil of tobacco and maize were higher under intercropping than monoculture (**Figure 5E**). The most abundant microbial genera were *Massilia* and *Gemmatimona*. The relative abundance of *Massilia* and *Gemmatimonas* in soil were lower in AMF inoculation treatments than non-inoculated treatments. The relative abundances of *Gemmatimonas* and *Sphingomonas* in the soil were higher under intercropping than monoculture (**Figure 5F**).

##### 3.3.2.2 Effects of AMF inoculation and planting pattern on the functions of microbial in rhizosphere soil of tobacco and maize

**Figure 6:**
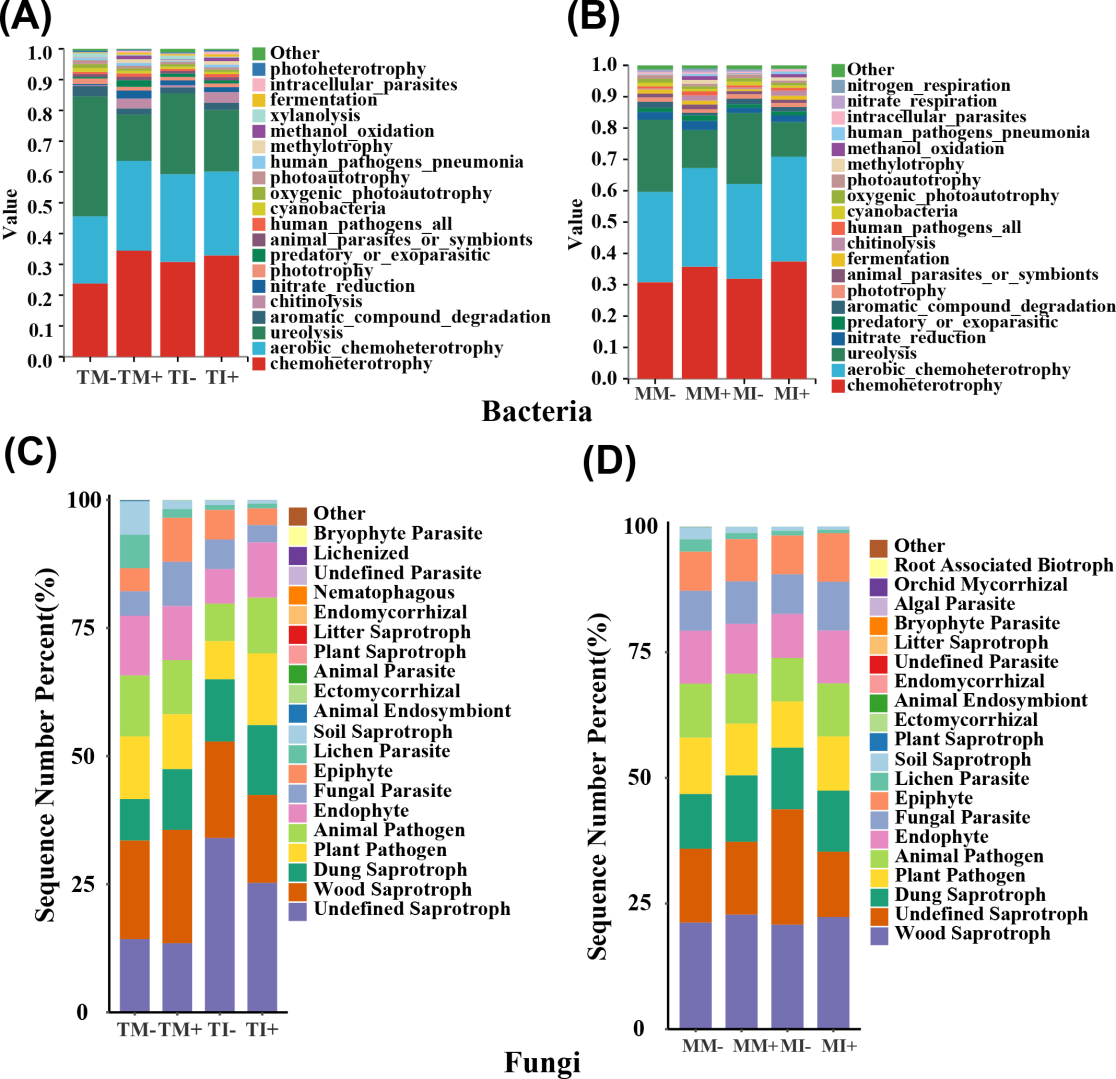
Comparison of the abundance predicted by microbial function, **(A, B)** are the prediction of bacterial function in tobacco and corn rhizosphere soil, respectively; **(C, D)** are the comparison of the predicted abundance of tobacco and corn rhizosphere soil fungi, respectively.

The FATROTAX database was used to predict the ecological functions of bacteria (Li et al., 2025), and a total of 60 functional categories were recovered. The main functions of bacteria in rhizosphere soil of tobacco and maize were chemoheterotrophy, urea degradation, and nitrate reduction. The relative abundance of the chemoheterotrophy, methylation, and methanol oxidation functions of bacteria in soil were significantly higher in AMF inoculation treatments than non-inoculated treatments. The relative abundances of chemoheterotrophy, chitin dissolution, and fermentation of bacteria in tobacco rhizosphere soil were significantly higher under intercropping than monoculture. Planting pattern and AMF inoculation significantly altered the relative abundance of urea degradation, aromatic compound degradation, and the photoautotrophic function of bacteria in tobacco rhizosphere soil (**Figure 6A**). The relative abundances of chemoheterotrophy, oxidative chemoheterotrophy, and aromatic compound degradation of bacteria in maize rhizosphere soil were significantly higher under intercropping than monoculture. The interaction between planting pattern and AMF had a significant effect on the relative abundance of bacteria in maize rhizosphere soil with four functions: chemoheterotrophy, xylan hydrolysis, oxidative chemoheterotrophy, and predation or ectoparasitism (*p* < 0.05) (**Figure 6B**).

The functions of fungi in different treatments were predicted based on the FUNGuild database (Zhan et al., 2025), and the trophic modes were pathotrophy, saprotrophy, and symbiotrophy. The relative abundances of animal endosymbiosis, lichen parasitism, plant pathogens, and soil humus functional fungi in tobacco rhizosphere soil were significantly lower in AMF inoculation treatments than non-inoculated treatments (*p* < 0.05) (**Figure 6C**), The relative abundances of endosymbiotic functional fungi were significantly increased in maize rhizosphere soil in AMF inoculation treatments than non-inoculated treatments, and they were also significantly higher under intercropping than monoculture in maize rhizosphere soil. The relative abundance of ectomycorrhizal functional fungi was significantly higher in tobacco rhizosphere soil under intercropping than monoculture (*p* < 0.05) (**Figure 6D**).

##### 3.3.2.3 Analysis of bacterial and fungal co-occurrence networks in rhizosphere soil of tobacco and maize

**Figure 7:**
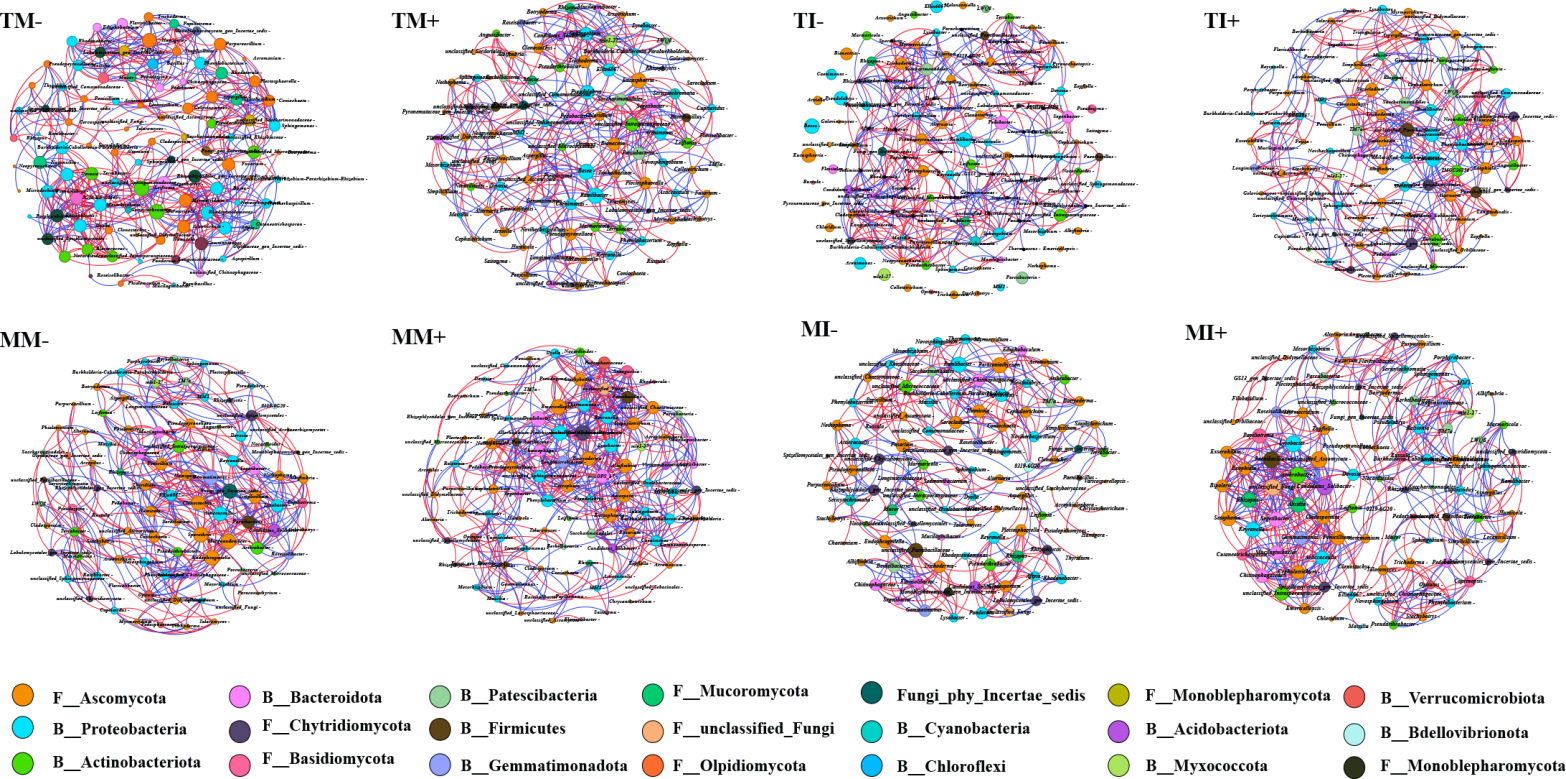
Bacterial and fungal co-occurrence networks in rhizosphere soil of tobacco and maize based on Spearman correlation analysis. Nodes represent the genera involved in the network, different colors of nodes represent different phyla, and links represent the relationships between nodes. The red line represents a significant positive correlation (Spearman’s correlation, r > 0.6, *p* < 0.05), and the blue line represents a negative correlation (Spearman’s correlation, r < -0.6, *p* < 0.05).

A co-occurrence network analysis of the 50 most abundant bacterial and fungal genera in the soil was performed (**Figure 7**; **Supplementary Table S2**). The results showed that the major nodes of bacteria and fungi in rhizosphere soil comprised 12 bacterial phyla and 9 fungal phyla. Ascomycota and Chytridiomycota were the major fungal phyla, and Proteobacteria and Actinobacteriota were the major bacterial phyla. The number of network nodes, clustering coefficients, and average path lengths were higher for co-occurrence networks under intercropping than monoculture. The number of network nodes and edges and the average degree of maize rhizosphere soil were higher in AMF inoculation treatments than non-inoculated treatments. These findings indicate that intercropping and AMF inoculation increased the complexity of the co-occurrence network structure of fungi and bacteria in soil. The proportion of positive correlations between bacteria and fungi in the soil was higher than negative correlations for AMF inoculation and the interaction of tobacco and maize roots, indicating that reciprocal relationships were stronger than competitive relationships.

#### 3.3.3 Effects of AMF and planting pattern on the metabolites of tobacco and maize

A total of 1,385 metabolites were detected in rhizosphere soil of tobacco and maize, and this included 13 types of metabolites, such as flavonoids, alkaloids, lipids, terpenoids, and phenolic acids. Flavonoids, lipids, terpenes, alkaloids, and phenolic acids were the major components of tobacco and maize root exudates (**Supplementary Figure S3A**). Principal component analysis (PCA) revealed a dense distribution of QC samples, as well as large variation and a high degree of aggregation in the metabolites of tobacco rhizosphere soil among different treatments (**Supplementary Figure S3B**). Large differences were observed between AMF inoculation treatments and non-inoculated AMF treatments in maize rhizosphere soil, suggesting that AMF inoculation had a strong effect on the metabolites in maize rhizosphere soil (**Supplementary Figure S3C**).

**Figure 8:**
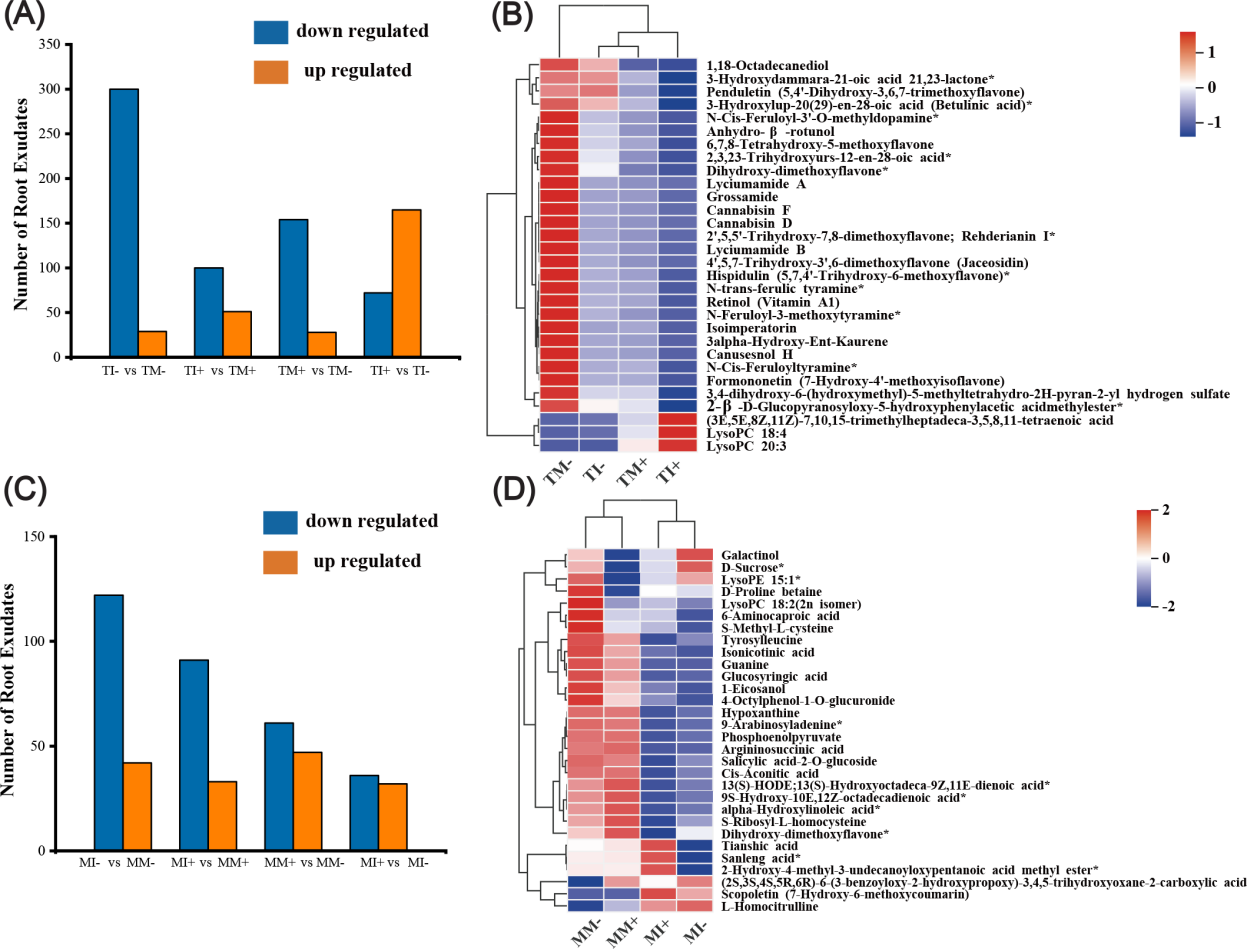
Identification and analysis of different metabolites in tobacco and maize rhizosphere soils. **(A, C)** are statistical charts of different metabolites in tobacco and maize root soil, respectively. **(B, D)** show the cluster analysis diagram of differential metabolites of tobacco and corn (Top30), respectively, and the color indicates the relative expression size of the metabolite in this group of samples.

Both interaction and inoculation of AMF decreased the amount of differentially expressed metabolites in the rhizosphere soil of tobacco and maize (**Figures 8A, C**). Analysis of root exudates with the top 30 VIP values of differential metabolites in soil showed that after AMF inoculation the main up-regulated root exudates of tobacco were lysophosphatidylcholine 18:4, lysophosphatidylcholine 20:3, and (3E,5E,8Z,11Z)-7,10,15-trimethylheptadeca-3,5,8,11-tetraenoic acid, while other metabolites were down-regulated under interplanting. Root exudates were down-regulated in tobacco rhizosphere soil under intercropping, with the exception of lysophosphatidylcholine 18:4 (**Figure 8B**). Compared with non-inoculated AMF treatments, maize root exudates were down-regulated in AMF inoculation treatments, with the exception of sanleng acid*, tianshi acid, and scopoletin. Compared with monoculture, maize root exudates were down-regulated under intercropping, with the exception of inositol galactoside, L-homocitrulline, D-sucrose*, and scopoletin (**Figure 8D**). This indicated that AMF inoculation and intercropping inhibited the synthesis of some DEMs of tobacco and maize roots.

**Figure 9:**
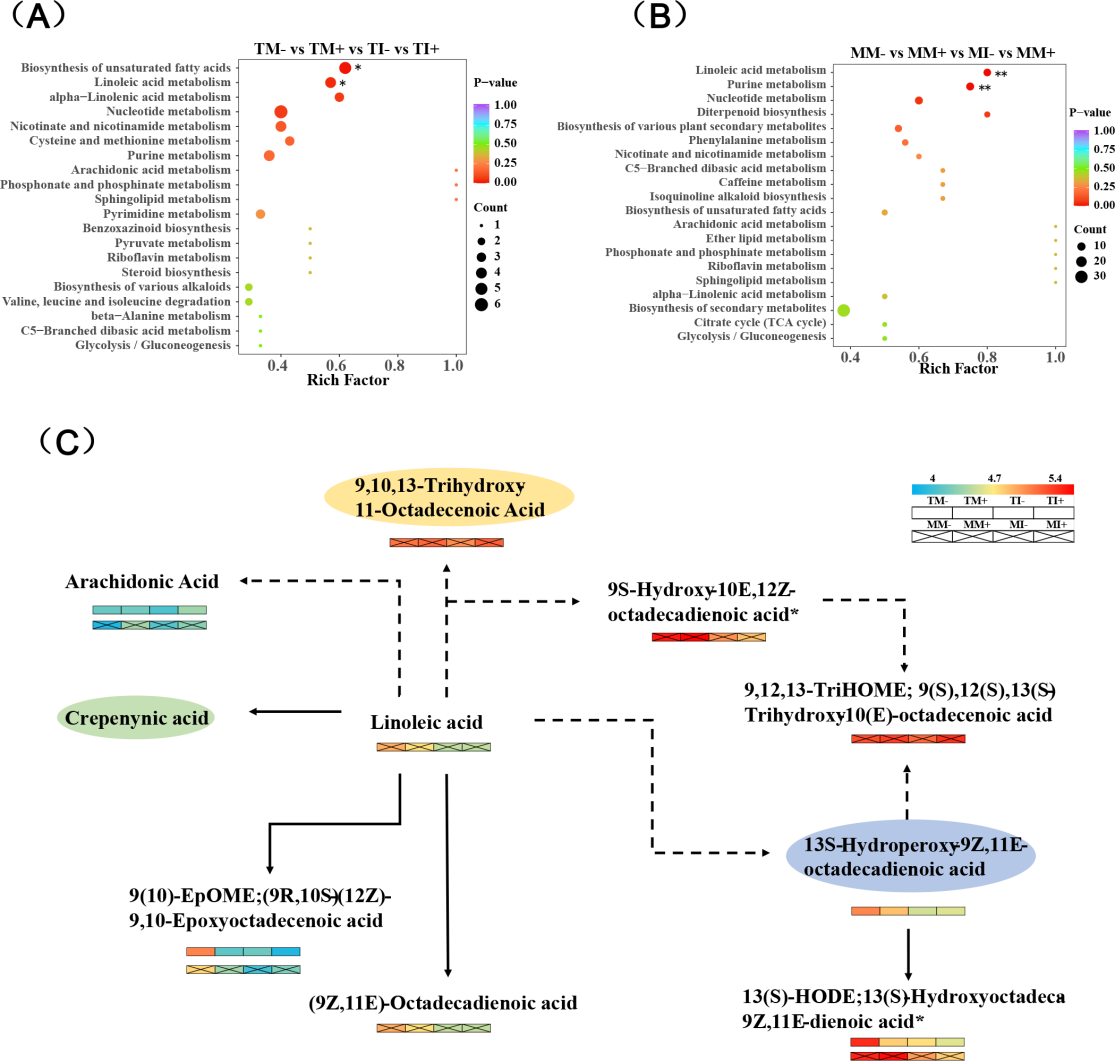
Important metabolic pathways of enrichment and differential metabolites in KEGG pathway. [**(A, B)** are KEGG pathway enrichment maps of tobacco and maize, respectively. **(C)** is the pathway with more enrichment among the selected key enrichment pathways (secondary metabolic pathways), the one without ellipse has significant difference, the one with ellipse has no significant difference, and the dashed line represents other pathways in the middle].

DEMs were significantly enriched in the metabolic pathways of secondary metabolites, including unsaturated fatty acids and linoleic acid in tobacco rhizosphere soil (**Figure 9A**), and DEMs of purine and linoleic acid metabolic pathways were significantly enriched in maize rhizosphere soil (*p* < 0.01) (**Figure 9B**). AMF inoculation and intercropping significantly affected the abundance of arachidonic acid, linoleic acid, (9R,10S)-(12Z)-9,10-epoxyoctadecenoic acid, 13S-hydroperoxy-9Z,11E-octadecadienoic acid, and 13(S)-Hydroxyoctadeca-9Z,11E-dienoic acid*.In particular, the abundance of arachidonic acid was significantly up-regulated, and the abundance of 13(S)-hydroxyoctadeca-9Z,11E-dienoic acid* was significantly down-regulated (**Figure 9C**).

### 3.4 The effects of soil agrochemical properties on microbial community diversity

An RDA was performed to assess the relationships between soil agrochemical properties and microbial. On the fungal community structure of tobacco rhizosphere soil, RDA1 and RDA2 explained 55.04% and 19.41% of the variation in the data, respectively; OM and AN were the main factors affecting the fungal community (**Supplementary Figure S4A**). In the analysis of the fungal community structure of maize rhizosphere soil, RDA1 and RDA2 respectively explained 49.01% and 27.3% of the community variation, pH, AK, and AN were the main factors affecting the fungal community (*p ≤* 0.01) (**Supplementary Figure S4B**). In the analysis of the microbiota community in tobacco rhizosphere soil, RDA1 and RDA2 explained 55.15% and 21.83% of the community variation, respectively, OM and pH were the main factors affecting bacterial community composition (*p* ≤ 0.05) (**Supplementary Figure S4C**). In the analysis of the microbial community in maize rhizosphere soil, RDA1 and RDA2 explained 54.25% and 25.5% of the community variation in the data, respectively. pH, AK, and AP were the main factors affecting the composition of the bacterial community (*p ≤* 0.01) (**Supplementary Figure S4D**).

**Figure 10:**
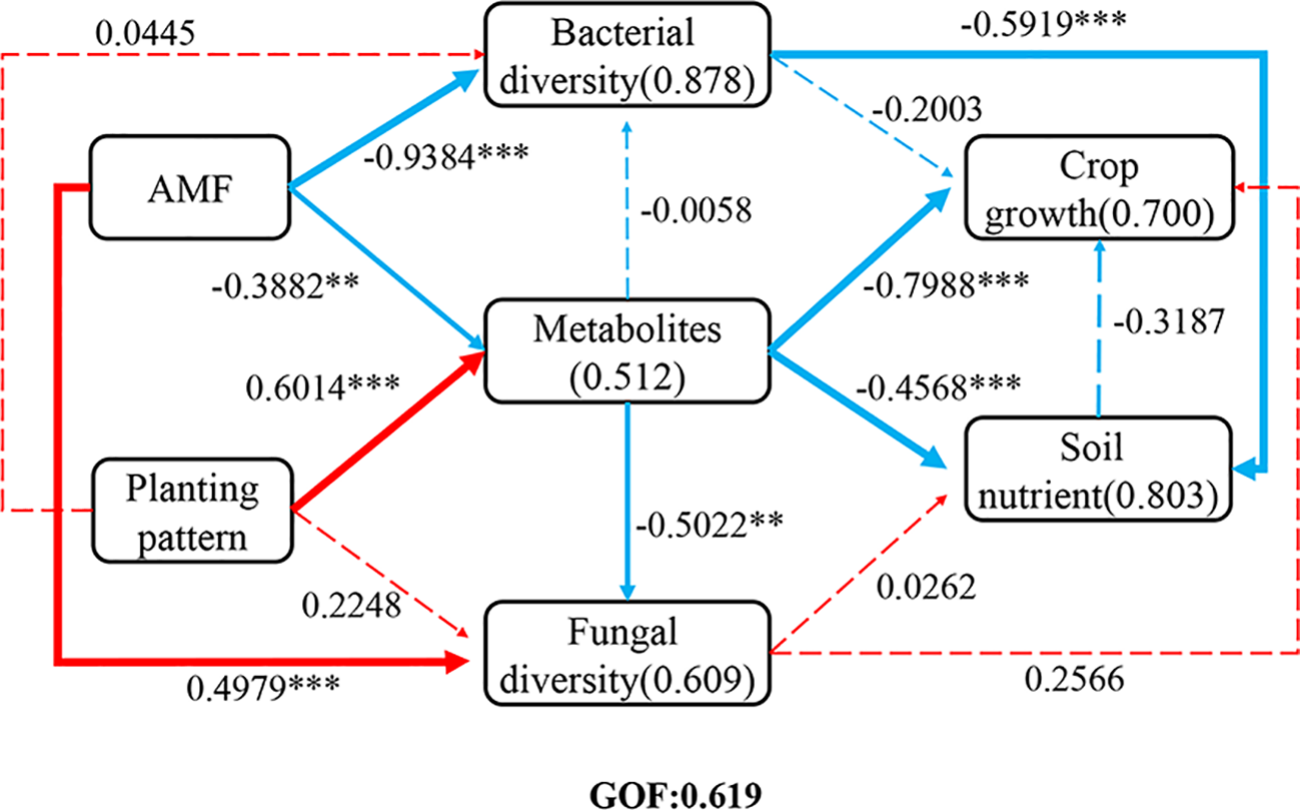
Partial least squares path model (PLS-PM). Direct and indirect effects of soil metabolite, bacterial and fungal community diversity on soil nutrients and crop growth under different planting patterns and AMF treatments. The width of the arrow is proportional to the significance of the direct effect, with dotted lines indicating non-significance and solid lines indicating significance. The red and blue arrows indicate the positive and negative flow of causality, respectively. The numbers on the lines represent the path coefficients, and the numbers in parentheses represent the variance R2 of the dependent variable interpreted by the model. Independent sample t-test for significant differences “* 0.01≤p<0.05, **0.001≤p<0.01, *** p<0.001”.

PLS-PM was used to analyze the direct and indirect effects of soil metabolites and bacterial and fungal community diversity on soil nutrients and plant growth under different planting patterns and AMF inoculation (**Figure 10**). The results showed that the model was well-fitted with a goodness-of-fit of 0.619. AMF inoculation had a significant negative effect on metabolites, fungal and bacterial community diversity in soils. Planting pattern had a significant positive effect on the metabolites in soil, and soil metabolites and microbiota diversity had a significant negative effect on nutrients. These results indicated that changes in metabolites and microbial diversity in soil had significant effects on soil nutrients and plant nutrient uptake under intercropping and AMF inoculation, and the microbial could regulate plant nutrient uptake and affect plant growth by improving soil nutrients.

## 4 Discussion

Intercropping with maize in the harvesting period of tobacco has been widely used in agricultural production, which is conducive to the sustainable development of tobacco and grain crops and helps to overcome the continuous cropping barrier of tobacco. tobacco and maize are typical mycorrhizal crops, and many studies have shown that their roots are colonized with a large amount of AMF (Wang et al., 2019). In this study, AMF inoculation increased the biomass of tobacco and maize and significantly improved leaf photosynthesis; intercropping increased the Pn of tobacco and maize and maize biomass but decreased tobacco biomass. Consistent with our findings, Sharma et al (Sharma and Kayang, 2017). found that AMF inoculation significantly increased the growth of *Camellia sinensis*, Zhu et al (Zhu et al., 2014). inoculated *Glomus versiforme* into *Robinia pseudoacacia*, which significantly enhanced the photosynthesis of plant seedlings. AMF inoculation can enhance the photosynthetic intensity of plants, promote light energy absorption and utilization efficiency, and thus significantly increase plant biomass (Yinli and Huili, 2021) (Li and Cai, 2021). found that AMF and biochar could increase the Pn of crops (Zou et al., 2019). found that the Pn was significantly higher following interactions among the roots of millet and peanut than monoculture, which may stem from improvements in light transmission and the photosynthetic capacity of leaves related to the planting pattern. In this study, rhizosphere interactions between maize and tobacco were observed during vegetative period. The root biomass of tobacco was lower under intercropping than monoculture, which probably stems from the fact that maize can acquire nutrients more effectively than tobacco during the vegetative period (Lan et al., 2010). Maize is intercropped during the harvesting period of tobacco in field production, and the competition for nutrients with maize promoted the yellowing and harvesting of tobacco (Tao et al., 2024).

AMF inoculation significantly increased the content of AN in tobacco and the activity of ACP, UE, and phytase in tobacco rhizosphere soil; intercropping significantly decreased the pH and ACP activity, increased phytase activity in the rhizosphere soil of tobacco, and enhanced the content of AN and AK and the activity of UE in maize rhizosphere soil. Previous studies have shown that AMF infestation of different plants promotes the formation of a Common Mycorrhizal Network (CMN), which can mediate the transport of nutrients and other resources (Wang et al., 2017). In the plant–AMF soil system, AMF provide N, P, and other nutrients to plants and simultaneously acquire Carbon source from roots (Smith and Smith, 2011). The formation of arbuscular mycorrhizae can activate P in the soil and promote P uptake by plant roots (Li et al., 2022b). Soil enzymes are involved in the catalysis of almost all reactions involved in organic metabolism. They provide energy for plants and microorganisms, recycle soil nutrients, and help maintain the nutrient and microbial balance of soil, they are thus direct indicators of the diversity of soil microbial functions (Liu et al., 2023b). Consistent with the findings, AMF inoculation has been shown to increase the enzymes activity related to the activation and transformation of soil C, N, and P (Jabborova et al., 2021). AMF can secrete organic acids such as citric acid, acetic acid, and oxalic acid, as well as ACP, which promotes the solubilization and activation of P in the soil, affects soil microorganisms, and increases the AP content in the soil (Tran et al., 2020). Intercropping with different plants can improve soil agrochemical properties and enzyme activities and promote nutrient uptake. The intercropping of maize with potato can significantly increase the activity of UE, ACP, and peroxidase in plant rhizosphere soil (Lu et al., 2023). In our study, however, intercropping with maize significantly reduced the activity of peroxidase and ACP in tobacco rhizosphere soil, probably because intercropping increased the content of AP in soil and alleviated the P starvation of microorganisms and plants, which reduced the secretion of ACP and led to decreases in the activity of ACP in soil (Yang et al., 2022). The physical and chemical properties of soil affect soil enzyme activity, temperature, moisture, pH, nutrient availability, fungal/bacterial ratios, and root exudates, all of which affect soil enzyme activities (Meng et al., 2020). We hypothesize that AMF inoculation and planting pattern indirectly regulate the activities of soil enzymes by influencing fungal and bacterial diversity in soil and root exudates (**Figure 10**).

Soil microorganisms can promote the utilization of nutrients by plants, and AMF colonization can affect the diversity of microorganisms in soil (Wang et al., 2019). In this study, AMF inoculation and intercropping increased the abundance of beneficial functional bacteria in soil, AMF inoculation increased the fungi abundance of *Penicillium* and bacterial abundance of *Massilia*, and the relative abundance of *Penicillium*, *Trichoderma*, *Blastomonas*, and *Sphingomonas* in soil was higher under intercropping than monoculture. *Penicillium* is a beneficial fungus that has been shown to be an effective biocontrol agent (Guo et al., 2024b); *Sphingomonas* and *Bacillus* are common plant growth-promoting organisms that promote plant growth by producing plant hormones (Sajjad et al., 2020; Patani et al., 2024). *Massilia* can solubilize and activate P, which can increase the content of soil nutrients (Zheng et al., 2017), and *Trichoderma* could effectively improve crop resistance, reduce plant diseases, and promote plant growth (Ruan and Liu, 2020). This suggests that AMF inoculation is beneficial to crop growth and promotes an increase in the abundance and functions of beneficial microorganisms associated with soil P nutrients (Liu et al., 2025), which further promotes the absorption and utilization of P nutrients by plants. Co-occurrence network analysis was used to reveal potential interactions between soil microorganisms (Jiao et al., 2022); positive correlations in the CMN may reflect species synergy or ecological niche overlap, whereas negative correlations may reflect interspecific competition and ecological niche segregation (Deng et al., 2016). Our FUNGuild analysis indicates that the synergistic benefits of AMF inoculation and intercropping are partly mediated by a fundamental functional reprogramming of the rhizosphere fungal community. Specifically, AMF inoculation in tobacco resulted in the regulation of the soil environment, characterized by a significant reduction in the relative abundance of plant pathogens and soil humus-degrading fungi (**Figure 6C**). This observation aligns with the concept of mycorrhiza-induced resistance (Xu et al., 2025) and suggests a reallocation of plant carbon toward the AMF symbiont (Wang et al., 2025). Concurrently, we observed a crop-specific enrichment of functional fungi: the maize rhizosphere was enriched with endosymbiotic fungi, whereas tobacco in the intercropping system uniquely exhibited an increase in ectomycorrhizal fungi (**Figures 6C, D**). This finding implies that intercropping with maize may unlock novel defensive and nutrient-mobilizing benefits for tobacco through the recruitment of ectomycorrhizal fungi (Sun et al., 2025b). Collectively, these functional shifts in the fungal community directly and indirectly supported plant health and nutrient acquisition. Intercropping and AMF inoculation increased the complexity of fungal and bacterial network structure in maize rhizosphere soil. Inoculation of AMF increased the complexity of bacterial and fungal networks in maize rhizosphere soil under intercropping, the opposite results were observed under monoculture. Consistent with our findings, AMF had a greater effect on the structure of bacterial communities in soil than fungal communities, AMF also significantly increased the abundance of beneficial bacteria and fungi in the soil (Hao et al., 2021). This mainly stemmed from the fact that plant mycorrhization by the exogenous AMF could affect soil microorganism biomass and diversity by regulating plant root exudates or carbohydrates in soil, especially the structure and communities of beneficial microorganisms and the abundance of bacteria and fungi (Yuan-Ying and Liang-Dong, 2007; Søren, 2008; He et al., 2012), which in turn, can promote plant growth (Liu et al., 2020).

Roots release various organic substances in soil, and root exudates are the major driver of plant–soil microorganism interactions (Ghatak et al., 2021; Li et al., 2022a). The root exudates of plants are often altered by different agricultural practices such as intercropping. Qin et al (Li et al., 2021). found that intercropping reduced the concentrations of oxalic acid and citric acid in maize root exudates in *Perilla*/maize intercropping of pot experiments. The significant increase in pathogen abundance is one of the key challenges associated with continuous cropping, when the abundance of pathogenic microorganisms increases in tobacco rhizosphere soil, the root exudates of tobacco also significantly increase (Guo et al., 2021). The abundance of L-proline is significantly up-regulated after AMF inoculation, and the expression of proline metabolism-related genes enhances the resistance of plants to environmental stress (Zhang et al., 2023). Isorhamnetin-7-O-glucoside* has been demonstrated to show peroxynitrite and DPPH-scavenging activity (Sue et al., 2002). In this study, the expression of isorhamnetin-7-O-glucoside* and coixol was up-regulated in tobacco rhizosphere soil after intercropping with maize. Coixol is produced by the plant of poaceae such as maize, and the content of coixol was increased when maize and other plants are intercropped, it has an antagonistic effect on pathogenic fungi (*e.g.*, *Fusarium*) (Narh et al., 2019; Acharya et al., 2021), which is consistent with the results of our study. Phenolic acids are closely related to plant growth and are the most important allelopathic autotoxins, the accumulation of phenolic acids is an important factor associated with continuous cropping (Saviranta et al., 2009). Some phenolic acids, such as benzyl β-primeveroside, 3-Hydroxycinnamic Acid*, Anisic acid-O-feruloyl glucoside and 3-aminosalicylic acid, were down-regulated after intercropping of tobacco and maize, and the down-regulation of these phenolic acids may alter the soil environment by affecting the structure of microbial communities (Yuxiang et al., 2019). Significantly enriched KEGG pathways included unsaturated fatty acid, linoleic acid, and purine metabolic pathways, the up-regulation of the unsaturated fatty acid and linoleic acid content may increase the stress resistance of plants (Wang et al., 2023). Therefore, AMF inoculation and rhizosphere interactions of maize and tobacco may enhance plant growth and increase the stress resistance of plants by regulating metabolites.

Plant characteristics and soil nutrients are important factors affecting soil microbial (Lin et al., 2015). The RDA results showed that the content of AK and pH were the main factors affecting rhizosphere metabolites. Zhang et al. found that there was a significant correlation between the type and content of root exudates and levels of AN, AP, and AK in intercropped soils (Zhang et al., 2024). The results of the PLS-PM analysis showed that AMF inoculation had a significant effect on metabolites and the diversity of fungi in rhizosphere soil, the planting pattern had a significant effect on metabolites in the soil, and soil metabolites and microbial diversity had significant negative effects on soil nutrients. Nardi et al (Nardi et al., 2002). confirmed that soil physical and chemical properties are closely correlated with the metabolic activities of microorganisms, and they hypothesized that soil metabolites affected the relative abundance of rhizosphere microorganisms and indirectly enhanced the soil environment by stimulating the growth and activity of microorganisms. 4-Nitrophenol is an aromatic compound that is degraded by some bacteria that use 4-nitrophenol as a sole C and N source (Laha and Petrova, 1997). The content of the fungal metabolite des-O-methyllasiodiplodin* is affected by the exogenous addition of methyl jasmonate (He et al., 2014). Elemol has strong antibacterial activity (Mevy et al., 2006). In the study, 10 metabolites, including 4-nitrophenol, coixol, and elemol, were positively correlated with the relative abundance of *Sphingobium*, *Berkelbacteria*, and *Candidatus_Solibacter* (**Supplementary Figure S4G**), and 13 metabolites, such as 4-nitrophenol, des-O-methyllasiodiplodin*, and palmitaldehyde, were negatively correlated with the relative abundance of *Fusarium*, *Rhizophlyctis*, and *Cladosporium* (**Supplementary Figure S4F**). Overall, root exudates play an important role in the formation of the rhizosphere microbial (Guo et al., 2024a), suggesting that planting pattern and AMF inoculation jointly regulated root exudates and thus affected the community of microorganisms in rhizosphere soil, which in turn affected plant growth and soil nutrients (Ma et al., 2022; Lu et al., 2024). From a practical perspective, our findings suggest that the co-application of AMF inoculation and tobacco-maize intercropping can be an effective strategy to enhance soil health and crop productivity. This integrated approach reduces reliance on chemical fertilizers by improving nutrient use efficiency and fosters soil microorganisms that can inhibit pathogens. Finally, it is important to acknowledge a limitation of this study. While we confirmed successful root colonization by the inoculated *F. mosseae* morphologically, our high-throughput sequencing approach did not specifically track its abundance or interactions within the broader soil microbial network. Future research we will employing AMF-specific molecular markers (e.g., AML2 primer set) which could provide deeper insights into the fate and competitive dynamics of the inoculated fungus in this intercropping context.

## 5 Conclusions

This study demonstrates that intercropping tobacco with maize and inoculating with AMF synergistically enhance crop growth by reshaping the rhizosphere soil environment. The key mechanisms uncovered include: 1) Modulation of the soil microbial community, increasing beneficial taxa like *Penicillium*, *Trichoderma*, and *Sphingomonas*; 2) Reprogramming of soil metabolite profiles, particularly down-regulating specific phenolic acids and enriching pathways for unsaturated and linoleic fatty acids linked to stress resistance; and 3) Improvement of soil nutrient availability and enzyme activities, leading to greater nitrogen, phosphorus, and potassium accumulation in plants. The main contribution of this work is the integrated perspective it provides on the “plant-AMF-soil microbiome-metabolite” network, revealing how agricultural practices can be leveraged to engineer a beneficial rhizosphere. A primary limitation was the reliance on morphological evidence for AMF colonization; future studies we will employ AMF-specific molecular techniques to precisely quantify the inoculated strain’s persistence and contribution, and should focus on validating these findings under field conditions, exploring the long-term dynamics of the established microbial communities, and investigating the potential of combining AMF with other beneficial microbes to further optimize the sustainability of the tobacco-maize intercropping system.

## Data Availability

The datasets presented in this study can be found in online repositories. The names of the repository/repositories and accession number(s) can be found in the article/**Supplementary Material**. Raw sequencing data were deposited in the NCBI Sequence Read Archive of https://www.ncbi.nlm.nih.gov/sra/ PRJNA1203072 (bacteria) and https://www.ncbi.nlm.nih.gov/sra/ PRJNA1203099 (fungi).
