# Supplementary material for: AMF-mediated rhizospheric interactions by soil microbiota and metabolites in intercropping of tobacco and maize to regulate the soil nutrients
Source: Front Plant Sci. 2025 Nov 27;16:1683474. doi: 10.3389/fpls.2025.1683474 (PMC12695787; doi:10.3389/fpls.2025.1683474)
Supplement: Supplementary file 1 [file Supplementaryfile1.docx]

**Supporting materials**

Table S1. Root AMF colonization rate of flue-cured corn under different planting patterns

| Group | Colonization rate of hypha | Colonization rate of mycelium | Arbuscular colonization rate | Vesicle colonization rate | Total colonization rate |
| --- | --- | --- | --- | --- | --- |
| TM+ | 70.33%±0.084a | 1.33%±0.003b | 14.33%±0.009b | 2.33%±0.013b | 85.00%±0.006b |
| TI+ | 55.33%±0.026a | 2.67%±0.012ab | 30.33%±0.110b | 3.00%±0.015b | 84.67%±0.009b |
| MM+ | 48.33%±0.071a | 3.00%±0.011ab | 58.67%±0.100a | 9.00%±0.025a | 86.67%±0.007ab |
| MI+ | 61.00%±0.061a | 7.00%±0.020a | 38.00%±0.031ab | 3.67%±0.012ab | 88.33%±0.007a |

Note: The data in the table are average ± standard error (n = 3), and different lowercase letters in the same column indicate statistically significant differences between processing (*p*<0.05)..

Table S2 Topological characteristics of symbiotic network of bacteria and fungi in rhizosphere soil of maize and flue-cured tobacco

| Index | TM- | TM+ | TI- | TI+ | MM- | MM+ | MI- | MI+ |
| --- | --- | --- | --- | --- | --- | --- | --- | --- |
| Modularity | 0.618 | 0.614 | 0.605 | 0.618 | 0.579 | 0.481 | 0.687 | 0.62 |
| Clustering Coefficient Metric | 0.566 | 0.557 | 0.524 | 0.581 | 0.626 | 0.556 | 0.632 | 0.622 |
| Graph Distance | 3.711 | 3.563 | 3.657 | 3.818 | 3.518 | 3.293 | 3.929 | 3.546 |
| Graph Distance | 10 | 9 | 8 | 9 | 9 | 8 | 9 | 8 |
| Graph Density | 0.1 | 0.084 | 0.053 | 0.08 | 0.1 | 0.104 | 0.074 | 0.104 |
| Average Degree | 9.653 | 8.613 | 6.744 | 7.714 | 9.608 | 10.202 | 7.163 | 10.202 |
| Positive correlation | 248 | 198 | 233 | 195 | 236 | 243 | 196 | 255 |
| Negative correlation | 225 | 202 | 202 | 183 | 230 | 262 | 155 | 250 |
| Nodes | 98 | 98 | 129 | 98 | 97 | 99 | 98 | 99 |


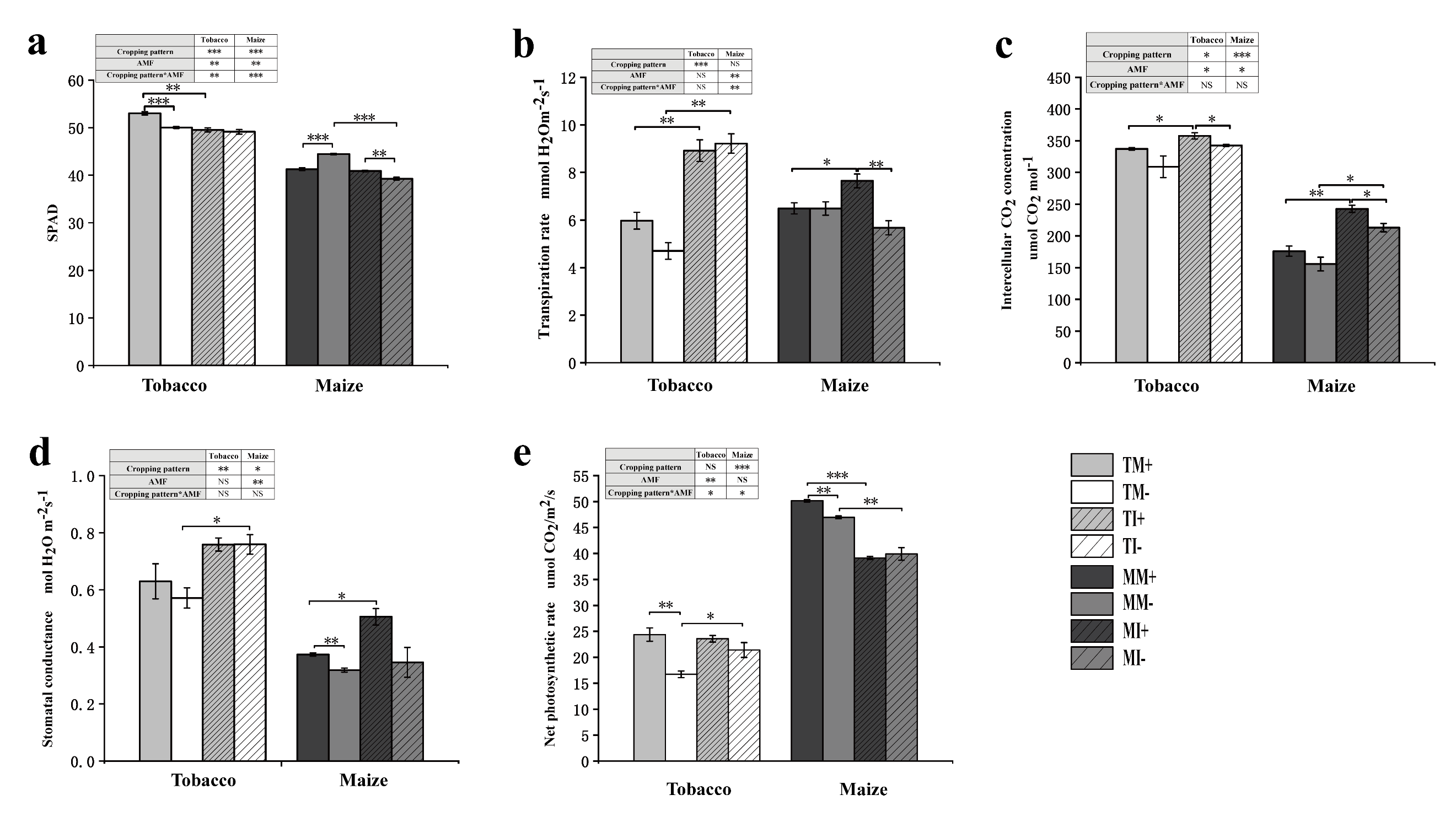


Fig. S1 Photosynthetic indices of flue-cured tobacco and maize in different treatment groups

Note: * indicates statistically significant difference between the two groups (*0.01≤p < 0.05, ** 0.001≤p < 0.01,*** p < 0.001, t test; n = 3). * In the table, the effects of planting methods and AMF on photosynthetic indices of flue-cured tobacco and maize were analyzed by two factors (* 0.01≤p < 0.05, ** 0.001≤p < 0.01,*** p < 0.001, bidirectional analysis of variance; N =3), the same below.


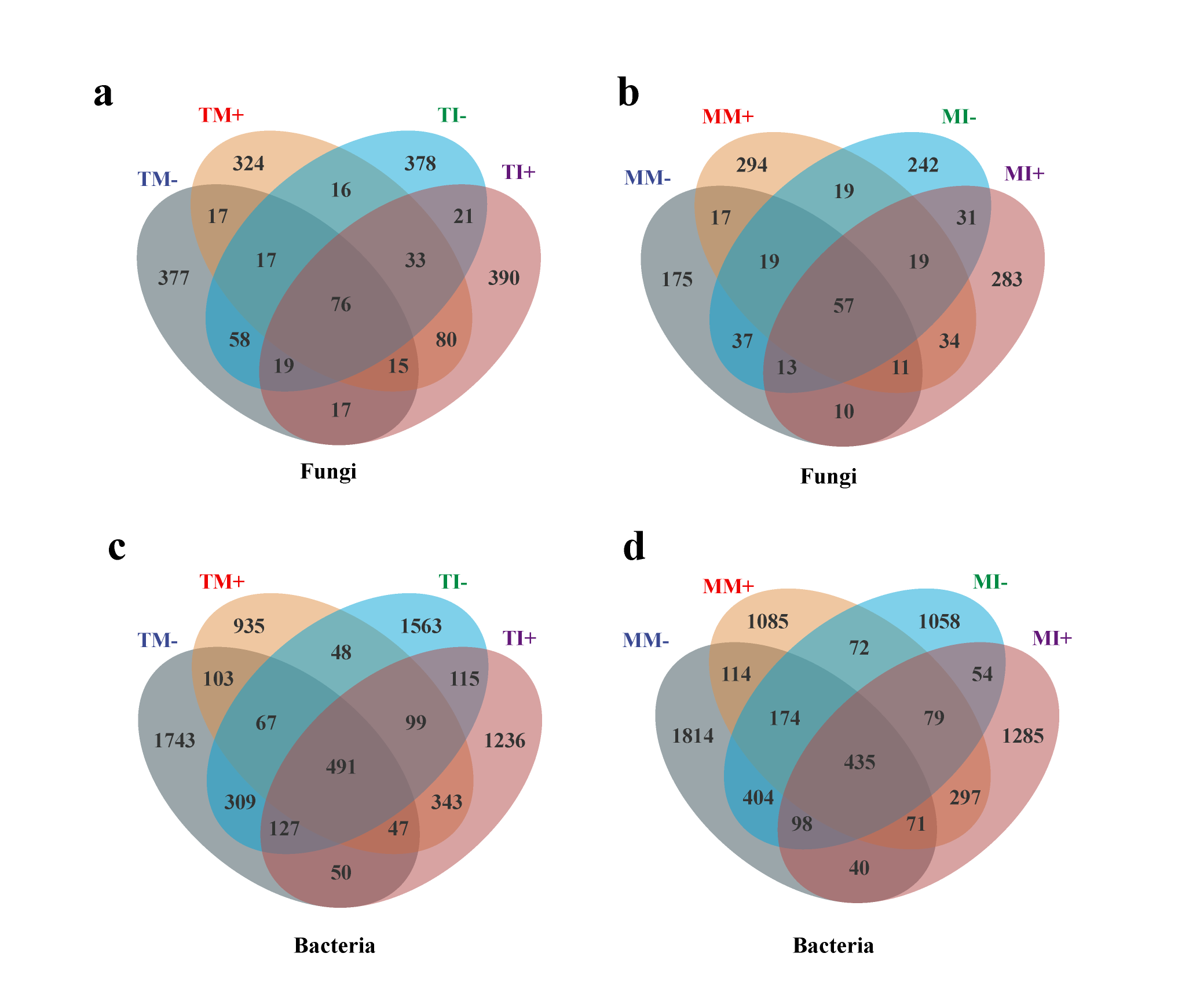


Fig. S2 Venn diagram of fungi (a, b) and bacteria (c, d) in rhizosphere soil of corn and flue-cured tobacco (ASV level)

Note: Each circle in the figure represents a sample, the number of overlapping circles represents the number of ASVs shared between samples, and the number of non-overlapping parts represents the number of unique ASVS of samples


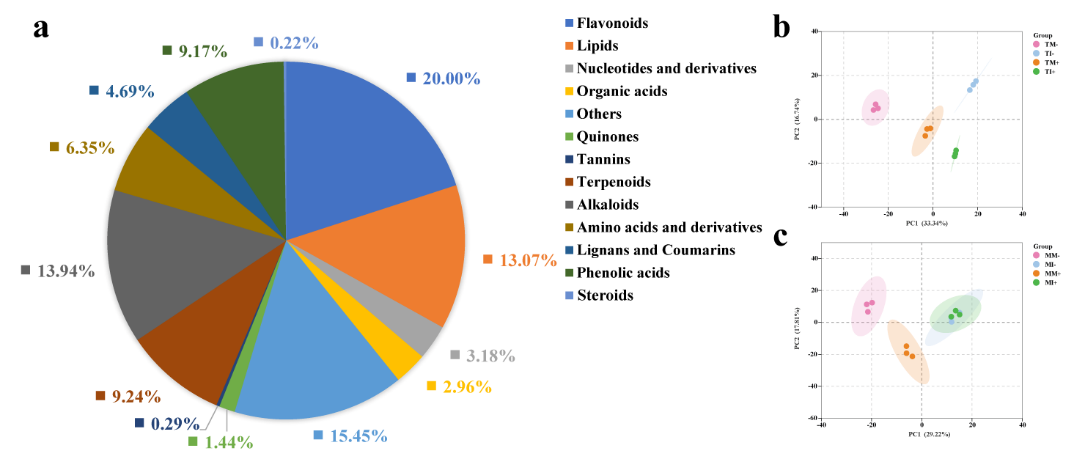


Fig. S3 Pie chart and PCA analysis of metabolites of flue-cured tobacco and maize

Note: Each color in Figure 2a represents a metabolite class, and the area of the color block indicates the proportion of the class.


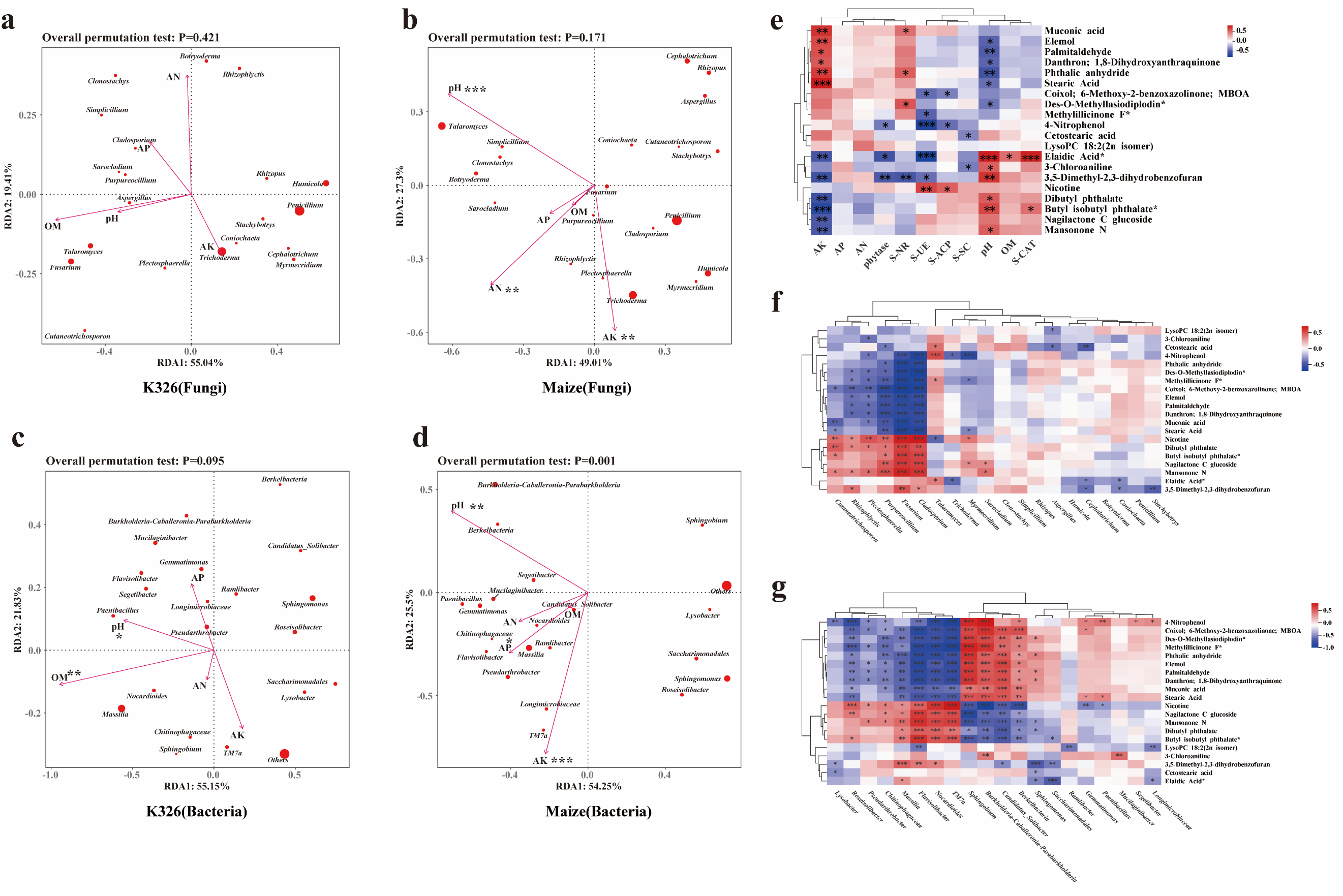


Fig. S4 Joint Analysis. Panels a, b, c, and d represent the redundancy analysis of flue-cured tobacco and maize rhizosphere soil fungi, bacteria, and soil environmental indices, respectively. Panel e illustrates the correlation between the primary differential metabolite and soil environmental indices. Panels f and g depict the correlations between soil fungi and bacteria and differential metabolites in key metabolic pathways, respectively. Significance levels: *** *p* < 0.001, ** *p* < 0.01, * *p* < 0.05.
